# Critically ill elderly patients (≥ 90 years): Clinical characteristics, outcome and financial implications

**DOI:** 10.1371/journal.pone.0198360

**Published:** 2018-06-01

**Authors:** Pierrick Le Borgne, Quentin Maestraggi, Sophie Couraud, François Lefebvre, Jean-Etienne Herbrecht, Alexandra Boivin, Baptiste Michard, Vincent Castelain, Georges Kaltenbach, Pascal Bilbault, Francis Schneider

**Affiliations:** 1 Emergency Department, Hautepierre Hospital, University Hospital of Strasbourg, Strasbourg, France; 2 INSERM (French National Institute of Health and Medical Research), UMR 1260, Regenerative NanoMedicine (RNM), Fédération de Médecine Translationnelle (FMTS), University of Strasbourg, Strasbourg, France; 3 Medical Intensive Care Unit and UMR 1121, Hautepierre Hospital, University Hospital of Strasbourg, Strasbourg, France; 4 Department of Public Health, University Hospital of Strasbourg, Strasbourg, France; 5 Department of Geriatrics, University Hospital of Strasbourg, Strasbourg, France; Azienda Ospedaliero Universitaria Careggi, ITALY

## Abstract

**Background:**

Patients aged over 90 are being admitted to intensive care units (ICUs) with increasing frequency. The appropriateness of such decisions still remains controversial due to questionable outcome, limited resources and costs. Our objective was to determine the clinical characteristics and outcome in elderly patients (≥ 90 years) admitted in a medical ICU, with an additional focus on medico-economic implications.

**Methods:**

We reviewed the charts of all patients (≥ 90 years) admitted to our ICU. We compared them with all other ICU patients (< 90 years), sought to identify ICU mortality predictors and also performed a long-term survival follow-up.

**Results:**

In the study group of 317 stays: median age was 92 years (IQR: 91–94 years); most patients were female (71.3%.). Acute respiratory failure (52.4%) was the main admission diagnosis; mean SAPS II was 55.6±21.3; half the stays (49.2%) required mechanical ventilation (duration: 7.2±8.8 days); withholding and withdrawing decisions were made for 33.4% of all stays. ICU and hospital mortality rates were 35.7% and 42.6% respectively. Mechanical ventilation (OR = 4.83, CI95%: 1.59–15.82) was an independent predictor of ICU mortality whereas age was not (OR = 0.88, CI95%: 0.72–1.08). Social security reimbursement was significantly lower in the study group compared with all other ICU stays, both per stay (13,160 vs 22,092 Euros, p< 0.01) and per day of stay (p = 0.03).

**Conclusion:**

Among critically ill elderly patients (≥ 90 years), chronological age was not an independent factor of ICU mortality. ICU care-related costs in this population should not be considered as a limiting factor for ICU admission.

## Background

According to the US Census Bureau, more than 1.87 million adults are 90 years or older (29% increase from 2000) [1]. As the population ages, intensive care units (ICUs) are confronted with increasing demand, with elderly patients now representing up to 20–30% of all admissions [2–3].

Firstly, studies suggest that physicians select patients based on chronological age albeit with considerable variations among centers [3–4]. Secondly, the geriatric specificities of these patients are not totally understood, and physicians’ knowledge concerning prognosis is not optimal. The initial triage process prior to ICU admission should be based on patient benefit, not determined solely by prognosis and comorbidities, but also accompanied by a functional approach and quality-of-life perspective. Most ICU studies focus on 30-day mortality, whereas 6-month or 1-year mortality seems more appropriate [5]. When a very old patient leaves the ICU alive, the rehabilitation process appears far more complex. Key factors contributing to a good recovery include frailty and comorbidity management [6–7].

Several questions remain open, such as the controversial benefit of ICU care for elderly patients [8]. Both ICU and in-hospital mortality rates remain high in critically ill elderly patients, with a large difference in outcome depending mainly on the motive for admission. Thus, surgical patients have a satisfactory outcome in contrast to medical patients who are at higher risk of death [9–10]. Moreover, many patients die soon after ICU discharge and few have a good recovery one year after discharge. The long-term recovery of functional status seems low, only one quarter return to baseline levels after 12 months [11].

End-of-life issues are critical in this population. Ambiguous directives make the management of such patients complex, highlighting the significance of proactively addressing goals [10,12].

In the current austere economic climate, cost-effectiveness considerations may also be included in medical decisions [13]. Concerning elderly patients, very few studies have addressed this particular implication [14]. It also appears that elderly patients receive less treatment in the ICU even after adjustment for severity of illness [15]. There are no current guidelines to assist the decision-making process, which leads to heterogeneity of practices [16].

The main objective of this study was to determine the clinical characteristics and outcome in elderly patients (≥ 90 years). In addition, we wanted to determine independent predictors of ICU mortality. Lastly, a particular focus was laid on medico-economic implications.

## Methods

### Settings

Our hospital is a teaching hospital with 2,200 beds, 179,000 stays, 70,000 patients in the Emergency Department (ED) and approximately 1,000 ICU admissions each year. In France, the development of out-of-hospital medical care allows direct ICU admission (from home or nursing home) for critically ill patients.

### Study population

The present study was a monocentric and retrospective analysis of collected data of all patients ≥ 90 years admitted to a 30-bed medical ICU (mean duration of stay: 9 days) between January 2000 and December 2015. If a patient was admitted to the ICU several times, this was considered as multiple stays and data were analyzed for each stay.

### Data collection

For each patient, we collected demographic information including age, sex, motive for admission and disposition at hospital discharge. The existence of a fatal disease was reflected by the McCabe score and the functional status was evaluated by the Knaus classification [17–18]. Otherwise, we used the Charslon comorbidity index, which has been shown to predict the one-year mortality [19]. Clinical data encompassed the primary diagnosis, the comorbidities, the need for ventilation and organ support (catecholamines, renal replacement therapy), the length of ICU and hospital stay, the discharge information as well as occurrence of withholding or withdrawing life support. A long-term survival follow-up was obtained for all patients by direct contact with them, their relatives or their general practitioner.

Severity of illness was assessed using the Simplified Acute Physiology Score II (SAPS II) [20]. ICU and in-hospital outcomes were analyzed and compared with those of all other ICU patients (< 90 years) during the study period and the French general population (≥ 90 years) [21]. Sub-group analysis was performed in order to compare clinical characteristics and ICU procedures, firstly, between ICU survivors and non-survivors and, secondly, according to three consecutive periods (2000–2004, 2005–2009, 2010–2015).

Finally, we studied medico-economic data and activity tarification. In our healthcare system, care-payers reimburse ICU stays to hospitals according to annual tariff rates fixed by law. The all-inclusive price includes both a basic rate according to homogeneous disease-stay fare (or case-mix fare) and a daily, but fixed, supplement related to the intensity of care whatever the disease treated. During the study period tariffs were stable or slightly increasing for a given case-mix from 2000 to 2010, but were progressively decreasing from 2011 to 2015 thanks to a policy of improving care-related costs. There was no specific adjustment for age in this reimbursement from the social security budget in relation to either part of the tariff.

### Ethics

This study was approved by the institution’s ethics review board (reference: AMK/BG/2015/2015-34).

### Statistical analysis

The descriptive analysis of the qualitative variables gives the frequency of each value and the cumulative frequency, and that of the quantitative variables, gives location parameters (mean, median, first and third quartiles), and dispersion parameters (standard deviation, variance, range and interquartile range). Normality of the distributions was checked using the Shapiro-Wilk or Kolmogorov-Smirnov test. The Kaplan-Meier method was used to determine survival rates. Comparisons between qualitative variables were made using Chi-squared test or Fisher’s exact test. Comparisons between quantitative and qualitative variables were made using the Student’s t-test (or ANOVA) or Wilcoxon’s test (or Kruskall-Wallis test). To estimate the independent predictors of ICU mortality, logistic regressions were performed. Multivariate analyses were done using variables statistically significant in bivariate analyses (p<0.1) and with age. A stepwise regression based on the AIC was performed with backward selection (and with age always included in the model). The significance level was set at 5%. All analyses were made with R 3.2.2 software.

## Results

### Clinical characteristics

During the study period a total of 16,210 stays were completed in our ICU. In 317 stays the patient was 90 years or older (1.96%) with 22 multiple stays (6.9%). Median age was 92 years, (IQR: 91–94) and the proportion of females was 71.3%. Most patients (39.8%) were admitted from the ED and about one third directly from home or nursing home by out-of-hospital services. Acute respiratory failure (52.4%) was the most frequent cause of admission followed by sepsis (11.4%). Mean ICU length of stay (LOS) was 7.0±8.0 days. Clinical characteristics of the study population can be consulted in **Table 1**.

**Table 1:** Characteristics of the study population.

General characteristics	All Stays n = 317	ICU Survivors n = 204	ICU Non-survivors n = 113	p value
Age (years), Median (IQR)	92 (91–94)	92 (91–94)	92 (91–94)	0.98^†^
Female, n (%)	226 (71.3)	151 (74.0)	75 (66.4)	0.15*
SAPS II, (Me±SD)	55.6±21.3	46.7±14.2	71.7±22.6	<0.01^††^
Glasgow score, Md (IQR)	14 (9–15)	14 (13–15)	12 (5–14)	<0.01^††^
**Comorbidities, n (%)**				
Cardiovascular diseases	297 (93.7)	195 (95.6)	102 (90.3)	0.06*
Chronic renal insufficiency	67 (21.1)	45 (22.1)	22 (17.2)	0.59*
Diabetes	50 (15.8)	34 (16.7)	16 (14.2)	0.56*
Neurodegenerative disease	54 (17)	32 (15.7)	22 (19.5)	0.69**
Cancer	57 (18)	35 (17.2)	22 (19.5)	0.61*
Respiratory diseases	75 (23.7)	47 (23.0)	28 (24.8)	0.73*
**Autonomy Scores, (Me±SD)**				
Charlson index	7.7±1.7	7.8±1.7	7.6±1.7	0.54^†^
Knaus score	2.18±0.65	2.14±0.63	2.25±0.69	0.17^†^
MacCabe score	1.3±0.5	1.3±0.5	1.3±0.5	0.89^†^
**Admission source, n (%)**				
Emergency Department	126 (39.8)	90 (44.1)	36 (31.9)	0.03*
Home	74 (23.3)	41 (20.1)	33(29.2)	0.04*
Nursing home	29 (9.1)	17 (8.3)	13 (11.5)	0.35*
Geriatrics	17 (5.4)	12 (5.9)	5 (4.4)	0.58*
Medical wards	43 (13.6)	33 (16.2)	10 (8.9)	0.07*
Surgical wards	20 (6.3)	8 (3.9)	12 (10.6)	0.02*
Post-operative	8 (2.5)	4 (2.0)	4 (3.5)	0.46**
**Diagnosis at admission**^a^**, n (%)**				
Cardiac arrest	28 (8.8)	8 (3.9)	20 (17.7)	<0.01*
Respiratory failure	166 (52.4)	118 (57.8)	48 (42.5)	<0.01*
Coma	30 (9.5)	15 (7.4)	15 (13.3)	0.08*
Sepsis	36 (11.4)	20 (9.8)	16 (14.2)	0.24*
Cardiovascular failure	33 (10.4)	20 (9.8)	13 (11.5)	0.63*
Trauma	4 (1.3)	3 (1.5)	1 (0.9)	1.00**
Metabolic	9 (2.8)	5 (2.5)	4 (3.5)	0.73**
Acute renal failure	27 (8.5)	18 (8.8)	9 (8.0)	0.79*
Intoxication	9 (2.8)	8 (3.9)	1 (0.9)	0.17**
Neurologic	11 (3.5)	7 (3.4)	4 (3.5)	1.00**
Gastrointestinal	13 (4.1)	7 (3.4)	6 (5.3)	0.56**
Miscellaneous	4 (1.3)	4 (2.0)	0	0.30**
**ICU LOS, days (Me±SD)**	7.0±8.0	6.2±5.6	8.5±11.0	0.43^††^

^a^ more than one diagnosis is possible.

ICU: intensive care unit, Me: mean, Md: median, IQR: interquartile range (25–75), SD: standard derivation, SAPS II: simplified acute physiology score, LOS: Length of stay.

* Pearson's Chi-squared test.

** Fisher's Exact Test.

^†^ t-test.

^††^ Wilcoxon rank sum test.

### ICU procedures

Almost half the study population (49.2%) underwent mechanical ventilation (MV) for an average of 7.2±8.8 days; 38.8% of all patients had exclusive non-invasive ventilation (NIV) for 2.9±2.6 days. Catecholamine support was applied in 47.6% of ICU stays and in 6.9% of stays a renal replacement therapy (RRT) was necessary. The decision to withhold or withdraw treatments was made for 33.4% of all stays, on average after 5.3 days (SD: 9.0 days) after ICU admission. ICU non-survivors had more MV and catecholamines (p<0.01) but less NIV (p = 0.02). Withholding and withdrawing therapy were used mainly in the non-survivors group (p<0.01), but later after admission than in survivors (6.1 vs. 1.9 days, p = 0.01). The list of ICU procedures is available in **Table 2**.

**Table 2:** Procedures in the ICU.

Procedures in the ICU, n (%)	All stays n = 317	ICU Survivors n = 204	ICU Non-survivors n = 113	p value
**Mechanical ventilation**	156 (49.2)	64 (31.4)	92 (81.4)	<0.01*
Days of MV (Me±SD)	7.2±8.8	6.2±5.3	7.8±10.6	0.19^††^
**Non-invasive ventilation**	123 (38.8)	89 (43.6)	34 (30.1)	0.02*
Days of NIV (Me±SD)	2.9±2.6	2.9±2.5	2.9±2.8	0.90^†^
**Catecholamines**	151 (47.6)	72 (35.3)	79 (69.9)	<0.01*
Epinephrin	42 (13.2)	7 (3.4)	35 (31)	<0.01*
Norepinephrin	97 (30.6)	43 (21.1)	54 (47.8)	<0.01*
Dobutamine	95 (30.0)	47 (23.0)	48 (42.5)	<0.01*
**Renal replacement therapy**	22 (6.9)	10 (4.9)	12 (10.6)	0.06*
Days with RRT (Me±SD)	4.7±8.0	1.8±1	7.2±10.3	1.00^††^
**Catheters**				
Arterial line	203 (64)	109 (53.4)	94 (83.2)	<0.01*
Central line	187 (59)	99 (48.5)	88 (77.9)	<0.01*
Swan-Ganz catheter	31 (9.8)	13 (6.4)	18 (15.9)	<0.01*
Urinary catheter	294 (92.7)	188 (92.2)	106 (93.8)	0.60*
**Antibiotics in the ICU**	218 (68.8)	140 (68.6)	78 (69.0)	0.94*
Bacteriemia	16 (5.1)	5 (2.5)	11 (9.7)	<0.01*
**Other procedures**				
Parenteral nutrition	36 (11.4)	17 (8.3)	19 (16.8)	0.02*
Blood transfusion sessions	39 (12.3)	18 (8.8)	21 (18.6)	0.01*
Chest tube	21 (6.6)	9 (4.4)	12 (10.6)	0.03*
Surgery	15 (4.7)	7 (3.4)	8 (7.1)	0.14*
**Withholding and Withdrawing**	106 (33.4)	20 (9.8)	86 (76.1)	<0.01*
Days after admission (Me±SD)	5.3±9.0	1.9 (2.4)	6.1 (9.7)	0.01^††^
Advanced end of life instructions (%)	54 (17.0)	16 (7.8)	38 (33.6)	<0.01**

ICU: intensive care unit, MV: mechanical ventilation, NIV: non-invasive ventilation, RRT: renal replacement therapy. Me: mean, Md: median, IQR: interquartile range (25–75), SD: standard deviation.

* Pearson's Chi-squared test.

** Fisher's Exact Test.

^†^ t-test.

^††^ Wilcoxon rank sum test.

### Clinical course and outcome

ICU mortality was 35.7% and in-hospital-mortality was 42.6% in the study population. ICU survivors and non-survivors did not differ significantly in terms of comorbidities. Non-survivors had higher severity, as illustrated by SAPS II (71.7 vs. 46.7, p<0.01). Patients admitted from the ED were more likely to survive the ICU stay (p = 0.03), contrary to patients admitted directly from home (p = 0.04) and respiratory failure was linked to better ICU outcome (p<0.01). There was no difference in terms of ICU LOS between survivors and non-survivors (p = 0.43).

Mechanical ventilation (OR = 4.83, CI95%: 1.59–15.82) and SAPS II (OR = 1.09, CI95%: 1.05–1.12) were independent predictors of ICU mortality in univariate and multivariate analysis. Withhholding and Withdrawing therapy (OR = 202.25, CI95%: 50.3–1145.2) and bacteremia were also associated with worse outcome. Other variables such as RRT, catecholamines, NIV and age (OR = 0.88, CI95%: 0.72–1.08) did not differ between ICU survivors and non-survivors (**Table 3**).

**Table 3:** Independent predictors of ICU mortality.

Predictors of ICU mortality	Odds Ratio	95%CI	p value
**Univariate analysis**			
Age	1.00	(0.89–1.11)	0.98
SAPS II	1.08	(1.06–1.09)	<0.01
Mechanical ventilation	9.51	(5.32–17.59)	<0.01
Non-invasive ventilation	0.56	(0.33–0.93)	0.02
Catecholamines	4.24	(2.53–7.22)	<0.01
Renal replacement therapy	2.30	(0.88–6.16)	0.06
Bacteriemia	4.27	(1.32–16.12)	<0.01
Withholding and Withdrawing	29.30	(15.90–56.52)	<0.01
**Multivariate analysis**			
Age	0.88	(0.72–1.08)	0.22
SAPS II	1.09	(1.05–1.12)	<0.01
Mechanical ventilation	4.83	(1.59–15.82)	<0.01
Non-invasive ventilation	2.27	(0.88–6.16)	0.10
Bacteriemia	4.60	(0.63–36.52)	0.14
Withholding and Withdrawing	202.25	(50.3–1145.2)	<0.01

ICU: intensive care unit, SAPS II: simplified acute physiology score, 95% CI: confidence interval.

**Fig 1:**
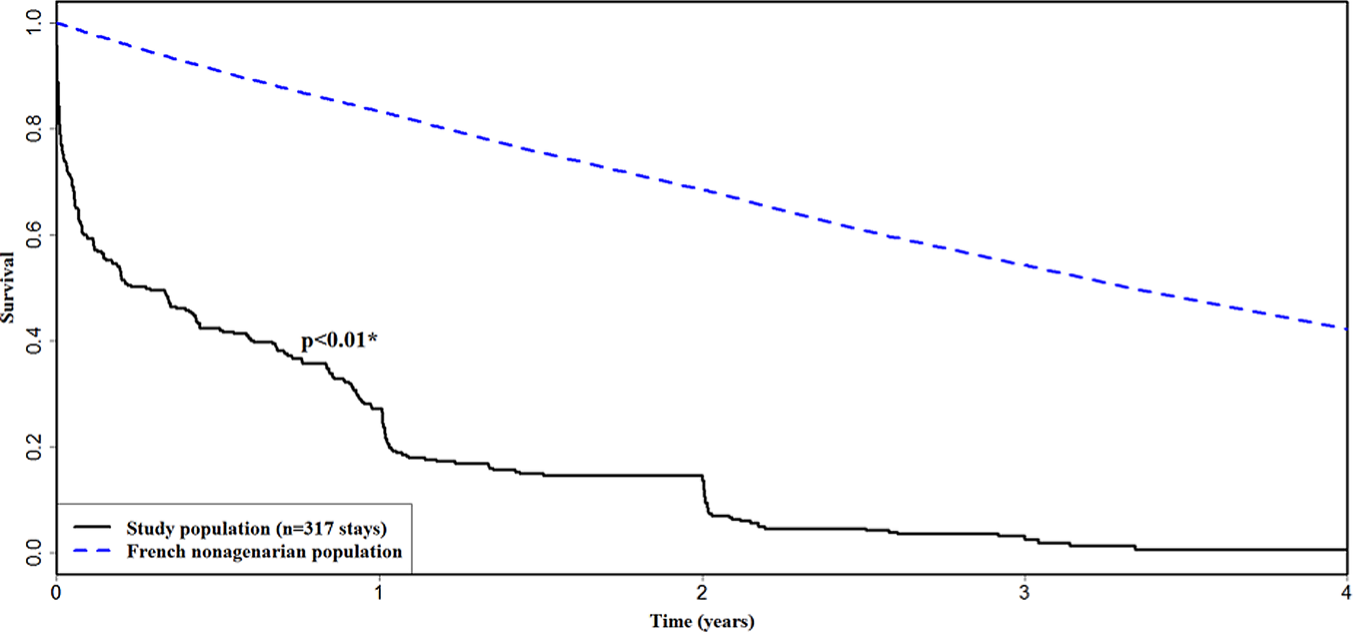
Survival from ICU admission. Kaplan-Meier survival curve of the study population in comparison with the French general poplation of similar age. Mortality data for the latter were obtained from INED [25]. ICU: intensive care unit. INED: institut national d’études démographiques. * Absolute Excess Risk test.

Most survivors were discharged to medical wards (45.7%) and geriatrics (14.8%). 90-day, 6-month and 1-year mortality were 47.3%, 55.8% and 69.7% respectively. We compared the study population with French population-based data of the same age group (**Fig 1**).

### Evolution of practices

We divided the study time into three periods (2000–2004, 2005–2009, 2010–2015) in order to compare patients characterictics, ICU procedures and survival. The proportion of elderly patients did not change over the time of the study (p = 0.26), nor did the ICU LOS (p = 0.76) and most procedures (**S1 and S2 Tables**); whereas severity and LOS increased significantly in younger patients from 2000 to 2015. However, we noticed a progressive increase in advanced end of life instructions which almost doubled from 2000 to 2015. Survival rates among elderly patients remained similar in all three periods (p = 0.27) (**S1 Fig**), while mortality in younger patients significantly decreased (data not shown).

### Medico-economic implications

We compared the financing of ICU stays by care-payers in the study population with a control group of all other stays of patients aged less than 90 years (n = 15,893) admitted to the ICU during the same period. The elderly population had a higher SAPS II score (p<0.01), but the ICU LOS was similar in both groups (p = 0.07). Social security reimbursement was significantly lower in the study group, both per stay (13,160 vs. 22,092 Euros, p<0.01) and per day of stay (3,305 vs. 4,332 Euros, p = 0.03). Over the previously defined study periods, we saw a divergence in the LOS and social security compensation between the study group and the control group. From 2000 to 2015, severity, LOS and reimbursements remained stable in the study population whereas in younger patients all three increased significantly (**Tables 4 and 5**).

**Table 4:** Comparison of social security conpensation between the study population (nonagenarians) and the control population over the same period (all ICU patients < 90 years old).

Medico-economic data	Control group n = 15893 (< 90 years)	Study group n = 317 (≥ 90 years)	p value
**All periods (2000–2015),** n = 16210 patients			
SAPS II	46.1±23.9	56±22.1	<0.01
ICU Length of stay (days)	9.8±21.6	6.3±8.7	0.07
Social Security retribution per stay (Euros)	22092±30772	13160±11070	<0.01
Social Security retribution per day of stay (Euros)	4332±8978	3305±3011	0.03

All data are Mean ±SD

SAPS II: simplified acute physiology score, SD: standard deviation.

**Table 5:** Comparison of care-related income between study population (nonagenarians) and all other patients (< 90 years old).

Periods of study	Control group n = 15893 (< 90 years)	Study group n = 317 (≥ 90 years)	p value
**2000–2004** (n = 5781 patients)	5672 (98.12)	109 (1.88)	** **
SAPS II	40.7±25.5	52.5±19.7	<0.01
ICU Length of stay (days)	8.8±24.8	6.4±8.0	0.08
Social Security retribution (Euros) per stay	16138±29025	12603±9088	0.85
Social Security retribution (Eur) per day of stay	3168±4375	2429±1513	0.79
**2005–2009 (**n = 4714 patients)	4609 (97.77)	105 (2.23)	
SAPS II	48.7±23.5	56.9±25.1	<0.01
ICU Length of stay (days)	10.7±22.1	6.3±9.1	<0.01
Social Security retribution (Eur) per stay	20852±33158	11912±10500	<0.01
Social Security retribution (Eur) per day of stay	3428±6715	3091±2325	0.01
**2010–2015** (n = 5715 patients)	5612 (98.20)	103 (1.80)	
SAPS II	49.3±21.3	58.7±21.0	<0.01
ICU Length of stay (days)	10.1±17.2	6.3±9.1	0.03
Social Security retribution (Eur) per stay	24246±28793	14548±12031	<0.01
Social Security retribution (Eur) per day of stay	5297±10940	3771±3778	0.30
**p value** (comparing the three study periods)			
SAPS II	<0.00001	0.16	
ICU Length of stay (days)	<0.00001	0.99	
Social Security retribution (Eur) per stay	<0.00001	0.22	
Social Security retribution (Eur) per day of stay	<0.00001	0.08	

All data are number (%) or Mean ±SD.

SAPS II: simplified acute physiology score, SD: standard deviation, Me: mean, Eur: euros, ICU: intensive care unit.

## Discussion

Most countries are now faced with the growing challenge related to global population ageing. In our region with good bed availability, ICU physicians have been admitting very old patients for a long time. Poor availability of ICU beds will require unbiased triage guidelines and it should ideally use different tools than in younger patients [22,23]. In this context, our data underscore two significant points: firstly, life expectancy of patients over 90 years old admitted to the ICU is limited to 3 years and secondly, the financial burden of critical care for these elderly patients is—on average—not on the increase compared with younger patients.

Our study showed a high ICU and in-hospital mortality. *Hwabejire et al*. [9] analyzed 474 trauma nonagenarians, with lower in-hospital mortality (9.5%) but higher 1-year mortality (40.5%). *Becker et al*. [24] examined 372 patients, the ICU and in-hospital mortality being 18.3% and 30.9% respectively. Other studies with elderly patients (mostly done with patients ≥ 80 years) demonstrated ICU mortality rates ranging from 15% to 50% [16]. In comparison, in-hospital mortality in younger ICU populations (45–65 years) ranges from 20% to 30% [25]. The comparability between all these studies is limited by the fact that outcome is dependent on the patient’s profile (medical or surgical, planned or unplanned admission) [26]. The fact that we had mainly medical and unplanned patients could explain our high mortality rate (likely leading to lower ICU costs); which is also being related to our liberal admission policy. Whereas age is identified as an independent risk factor for ICU mortality in non-selected populations, comorbidities, frailty and severity of illness appear to be more important risk factors than age itself in an elderly population [27].

ICU physicians also suggest that end-of-life decisions are not adequately weighed in this population with a poor short-term outcome [4]. We have observed an increasing number of end-of-life decisions over time in our study, which may explain why the ICU length of stay did not increase in the study population (≥ 90 years), yet it increased in younger patients. In fact, a high proportion of deaths in critically ill elderly patients follow a withdrawal of life-sustaining therapy. Furthermore, it is fundamental to assess the patient’s opinion, especially as the elderly are often reluctant to undergo life-sustaining treatments [12]. A recent study reported that only 13% had been asked about their willingness to be admitted to the ICU [28].

Studies focused on very elderly patients (≥ 90 years) admitted to ICUs are scarce. *Becker et al*. [24] have detected age, the need for vasopressors and renal impairment as independent factors of bad outcome within the ICU, while elective surgery did not negatively impact outcome. In our study, chronological age was not *per se* a factor of bad prognosis and this was stable throughout the study period. In contrast, the need for MV was independently associated with poor prognosis. The influence of NIV on outcome in our study population remains unclear, since it looked protective only in univariate analysis. Given the fact that it can easily be performed elsewhere than in the ICU, our data suggest it be used without limit.

Most elderly patients are treated in ICUs at heavy costs, in cooperation if necessary with organ specialists. Few of them are visited by a geriatrician despite recent data suggesting a need of such involvement [29]. The holistic approach of geriatricians could lead to better decision-making, help in the triage process and remain a key factor in post-ICU care. Specific geriatric ICUs are exceedingly rare [30]. Combining a multidimensional and interdisciplinary approach to coordinate treatments with the recovery process could be an efficient way of treating approximatively half the nonagenarians studied who did not require respiratory support.

Limited resources, costs and controversial benefit are currently the main reasons for the debate regarding ICU access for elderly patients [8,13–14]. It has been demonstrated that these patients receive lower treatment intensity and less life-sustaining treatment than younger patients even after adjustment for severity of illness [15]. Overall, in our study, the total amount of money dedicated to elderly patients is significantly lower than that for younger patients. This data suggests that elderly patients undergoing intensive care do not constitute a heavier healthcare burden. Thus, in our economic model, cost of stay cannot be considered as an obstacle to admission in ICUs. Our results suggest that ICU costs have to be studied in line with a close to 0% life expectancy 3 years after ICU admission. Appropriateness of care should be the main priority, as it is for younger patients in which a 3-year life expectancy is not an obstacle to ICU care. These are several reasons why guidelines need to be published with a view to clarifying admission criteria for elderly patients [16].

Our results should be interpreted with caution on account of several limitations. Firstly, our single-center study provides findings that may not be transposable to settings where ICU availability is different. Secondly, our follow-up did not provide insights into functional status after discharge and assessment of frailty which is now used to evaluate outcome [6–7]. Thirdly, there is no estimate of costs associated with overall hospital stay nor post-hospital healthcare utilisation, which may be significant in this very old population. Finally, further studies on long-term outcome are needed [16].

## Conclusion

Critically ill elderly patients (≥ 90 years) constitute a fast-expanding subgroup proposed for ICU admission. Until now, some physicians have been reluctant to admit them, mostly because of high in-hospital mortality; however prognosis is not as poor as often perceived. Our data suggests that chronological age is not a viable exclusion criterion for ICU admission, but rather that elderly patients should benefit of equitable access to ICUs. The present study adds to our understanding that there is no real financing issue regarding ICU care in patients over 90 years old.

## Supporting information

S1 Table Comparison of general characteristics of the study population according to the three consecutive periods of the study. (DOCX)

S2 Table Comparison of ICU procedures according to the three consecutive periods of the study. (DOCX)

S1 Fig Kaplan-Meier survival curves for the three periods of the study. (TIF)

## Abbreviations

ICU: intensive care unit
ED: Emergency Department
OR: odds ratio
SAPS II: Simplified Acute Physiology Score II
NIV: non-invasive ventilation
RRT: renal replacement therapy
