# Supplementary material for: Critically ill elderly patients (≥ 90 years): Clinical characteristics, outcome and financial implications
Source: PLoS One. 2018 Jun 1;13(6):e0198360. doi: 10.1371/journal.pone.0198360 (PMC5983531; doi:10.1371/journal.pone.0198360)
Supplement: S1 Table — (DOCX) [file pone.0198360.s001.docx]

**S1 Table**

| **General characteristics** | **2000-2004**  **n=109** | **2005-2009**  **n=105** | **2010-2015**  **n=103** | **p value** |
| --- | --- | --- | --- | --- |
| Female, n (%) | 70 (64.2) | 83 (79.1) | 73 (70.9) | 0.06^*^ |
| Age (years) (Mean±SD) | 92.4±1.9 | 92.9±2.1 | 92.6±2.4 | 0.32^†^ |
| SAPS II (Me±SD) | 51.4±17.5 | 56.5±25.0 | 59.3±20.3 | 0.03^††^ |
| Organ dysfunction (Me±SD) | 1.6±0.6 | 1.6±0.8 | 1.7±0.7 | 0.84^††^ |
| Glasgow score, Md (IQR) | 14 (11-15) | 14 (10-15) | 14 (8-15) | 0.59^†^ |
| **Autonomy scores ((Me±SD)** |  |  |  |  |
| Charlson index | 7.8±1.7 | 7.7±1.7 | 7.6±1.7 | 0.64^†^ |
| McCabe score | 1.3±0.5 | 1.4±0.5 | 1.3±0.5 | 0.12^†^ |
| Knaus score | 2.3±0.6 | 2.1±0.7 | 2.1±0.7 | <0.01^†^ |
| **Comorbidities, n (%)** |  |  |  |  |
| Cardiovascular diseases | 103 (94.5) | 94 (89.5) | 100 (97.1) | 0.07^*^ |
| Chronic renal insufficiency | 18 (16.5) | 21 (20.0) | 28 (27.2) | 0.15^*^ |
| Diabetes | 13 (11.9) | 18 (17.1) | 19 (18.4) | 0.38^*^ |
| Neurodegenerative disease | 15 (13.8) | 17 (16.2) | 22 (21.4) | 0.33^*^ |
| Cancer | 21 (19.3) | 22 (20.1) | 14 (13.6) | 0.35^*^ |
| Respiratory diseases | 28 (25.7) | 20 (19.0) | 27 (26.2) | 0.40^*^ |
| **Admission source, n (%)** |  |  |  |  |
| Emergency Department | 48 (44.0) | 45 (42.9) | 33 (32.0) | 0.15^*^ |
| Home | 25 (22.9) | 26 (24.8) | 23 (22.3) | 0.83^*^ |
| Nursing home | 13 (11.9) | 11 (10.5) | 5 (4.9) | 0.15^*^ |
| Geriatrics | 5 (4.6) | 5 (4.8) | 7 (6.8) | 0.73^*^ |
| Medical wards | 18 (16.5) | 8 (7.6) | 17 (16.5) | 0.09^*^ |
| Surgical wards | 0 | 7 (6.7) | 13 (12.6) | <0.01^*^ |
| Post-operative | 0 | 2 (1.9) | 6 (5.8) | 0.01^**^ |
| **Diagnosis at admission^a^, n (%)** |  |  |  |  |
| Cardiac arrest | 6 (5.5) | 9 (8.6) | 13 (12.6) | 0.19^*^ |
| Respiratory failure | 61 (56.0) | 58 (55.2) | 47 (45.6) | 0.25^*^ |
| Coma | 13 (11.9) | 7 (6.7) | 10 (9.7) | 0.42^*^ |
| Sepsis | 6 (5.5) | 12 (11.4) | 18 (17.5) | 0.02^**^ |
| Cardiovascular | 13 (11.9) | 5 (4.8) | 15 (14.6) | 0.06^*^ |
| Trauma | 0 | 1 (1.0) | 3 (2.9) | 0.08^**^ |
| Metabolic | 2 (1.8) | 4 (3.8) | 3 (2.9) | 0.65^**^ |
| Acute renal insufficiency | 9 (8.3) | 7 (6.7) | 11 (10.7) | 0.58^*^ |
| Intoxications | 4 (3.7) | 3 (2.9) | 2 (1.9) | 0.91^**^ |
| Neurologic | 0 | 1 (1.0) | 10 (9.7) | <0.01^**^ |
| Gastrointestinale | 3 (2.8) | 6 (5.7) | 4 (3.9) | 0.56^**^ |
| Miscellaneous | 0 | 3 (2.9) | 1 (1.0) | 0.17^**^ |
| **Length of stay, days (Me±SD)** | 7.0±7.5 | 6.9±8.2 | 7.1±8.4 | 0.78^††^ |

SAPS II: simplified acute physiology score, SD: standard deviation, IQR: interquartile range (25-75), Me: mean, Md: median, ^a^ more than one diagnosis is possible.

^*^ Pearson's Chi-squared test, ^**^ Fisher's Exact Test, ^†^ t-test, ^††^ Wilcoxon rank sum test
