# Supplementary material for: Critically ill elderly patients (≥ 90 years): Clinical characteristics, outcome and financial implications
Source: PLoS One. 2018 Jun 1;13(6):e0198360. doi: 10.1371/journal.pone.0198360 (PMC5983531; doi:10.1371/journal.pone.0198360)
Supplement: S2 Table — (DOCX) [file pone.0198360.s002.docx]

**S2 Table**

| **Procedures in the ICU, n(%)** | **2000-2004**  **n=109** | **2005-2009**  **n=105** | **2010-2015**  **n=103** | **p value** |
| --- | --- | --- | --- | --- |
| **Mechanical ventilation** | 48 (44.0) | 53 (50.5) | 55 (53.4) | 0.38^*^ |
| Average days with MV (±SD) | 7.8±8.5 | 6.4±8.7 | 7.4±9.3 | 0.69^†^ |
| **Non-invasive ventilation** | 46 (42.2) | 42 (40.0) | 35 (34.0) | 0.45^*^ |
| Average Days with NIV (±SD) | 3.0±2.3 | 2.6±3.2 | 3.0±2.0 | 0.12^††^ |
| **Catecholamines** | 46 (42.2) | 47 (44.8) | 55 (53.4) | 0.36^*^ |
| Epinephrine | 17 (15.6) | 13 (12.4) | 12 (11.7) | 0.66^*^ |
| Norepinephrine | 33 (30.3) | 25 (23.8) | 39 (37.9) | 0.09^*^ |
| Dobutamine | 30 (27.6) | 34 (32.4) | 31 (30.1) | 0.74^*^ |
| **Renal replacement therapy** | 10 (9.2) | 7 (6.7) | 5 (4.9) | 0.46^*^ |
| Average Days with RRT (±SD) | 3.7±4.3 | 4.1±7.9 | 7.6±13.7 | 0.51^††^ |
| **Catheters, n(%)** |  |  |  |  |
| Arterial line | 77 (70.6) | 62 (59.0) | 64 (62.1) | 0.19^*^ |
| Central line | 63 (57.8) | 57 (54.3) | 67 (65.0) | 0.27^*^ |
| Swan-Ganz catheter | 12 (11.0) | 7 (6.7) | 12 (11.7) | 0.42^*^ |
| Urinary catheter | 102 (93.6) | 98 (93.3) | 94 (91.3) | 0.78^*^ |
| Patients given antibiotics | 71 (65.1) | 67 (63.8) | 80 (77.7) | 0.06^*^ |
| **Other procedures, n(%)** |  |  |  |  |
| Parenteral nutrition | 10 (9.2) | 12 (11.4) | 14 (13.6) | 0.60^*^ |
| Blood transfusion sessions | 12 (11.0) | 11 (10.5) | 20 (19.4) | 0.11^*^ |
| Chest tube | 3 (2.8) | 10 (9.5) | 8 (7.8) | 0.12^*^ |
| Surgery | 1 (0.9) | 8 (7.6) | 6 (5.8) | 0.04^**^ |
| **Withholding and Withdrawing, n(%)** | 37 (33.9) | 29 (27.6) | 40 (38.9) | 0.23^*^ |
| Average days after admission (±SD) | 4.6±8.6 | 6.4±9.4 | 5.1±9.2 | 0.73^†^ |
| Advanced end of life instructions | 15 (13.8) | 13 (12.4) | 26 (25.2) | 0.03^*^ |

ICU: intensive care unit, Me: mean, MV: mechanical ventilation, NIV: non-invasive ventilation, RRT: renal replacement therapy, SD: standard deviation.

^*^ Pearson's Chi-squared test, ^**^ Fisher's Exact Test, ^†^ t-test, ^††^ Wilcoxon rank sum test
