# Supplementary figures and images for: Critically ill elderly patients (≥ 90 years): Clinical characteristics, outcome and financial implications
Source: PLoS One. 2018 Jun 1;13(6):e0198360. doi: 10.1371/journal.pone.0198360 (PMC5983531; doi:10.1371/journal.pone.0198360)

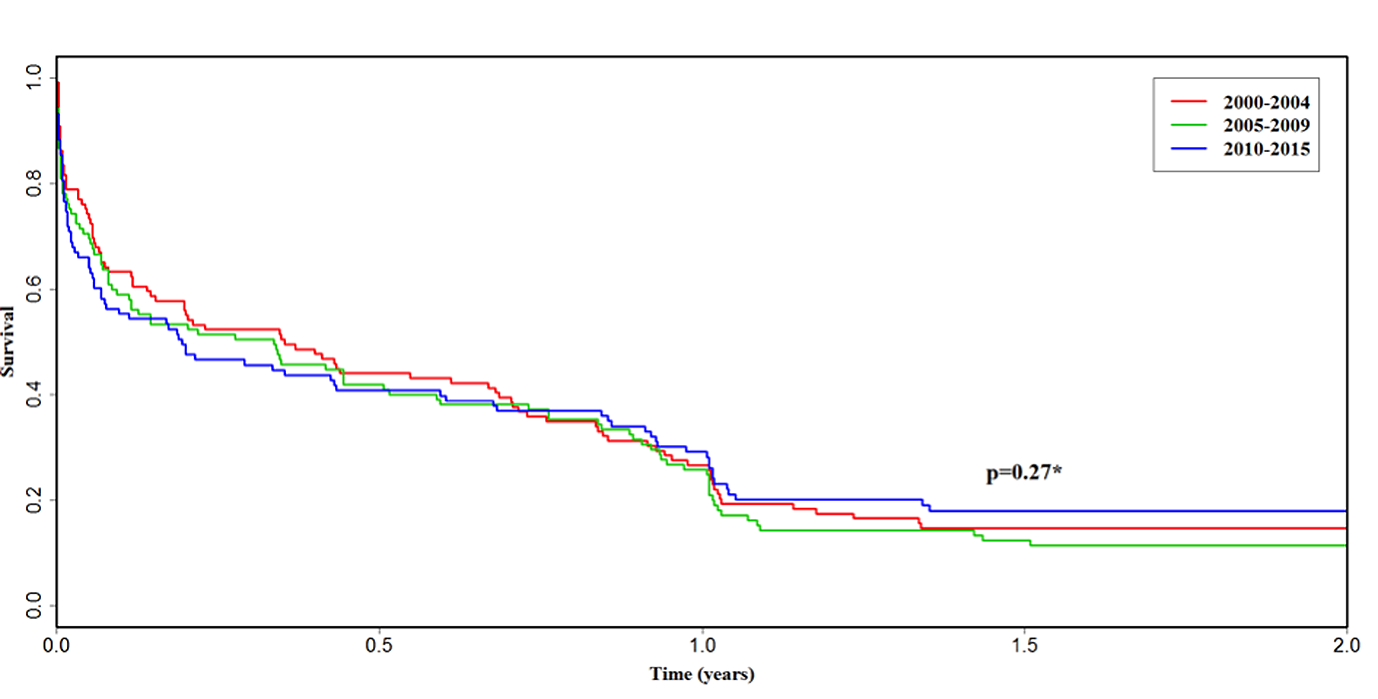

Supplement: S1 Fig — (TIF) [file pone.0198360.s003.tif]
